# B‐Site Multi‐Element Micro‐Doping Engineered Air Electrodes With Fast Oxygen Kinetics for Reversible Protonic Ceramic Electrochemical Cells

**DOI:** 10.1002/advs.78109

**Published:** 2026-09-30

**Authors:** Wenkai Yang, Yue Bao, Timileyin Aworinde, Linfeng Yu, Shaikh Abdullah, Lakshya Mathur, Daofan Wang, Lourdes F. Vega, Chuancheng Duan, Sivaprakash Sengodan

**Affiliations:** ^1^ Department of Mechanical and Nuclear Engineering Khalifa University Abu Dhabi UAE; ^2^ Research and Innovation Center on CO_2_ and Hydrogen (RICH) Khalifa University Abu Dhabi UAE; ^3^ Department of Chemical Engineering University of Utah Salt Lake City Utah USA; ^4^ Department of Chemical and Petroleum Engineering Khalifa University Abu Dhabi UAE; ^5^ Energy Institute Khalifa University Abu Dhabi UAE

**Keywords:** oxygen vacancy, perovskite, redox, reversible protonic ceramic electrochemical cells

## Abstract

Reversible protonic ceramic electrochemical cells (R‐PCECs) provide a promising route for integrated power generation and hydrogen production, yet their practical operation and commercialization remain constrained by sluggish and stability‐sensitive air electrode reactions at intermediate temperatures. Here, we employ a multi‐element micro‐doping strategy for designing layered double perovskite air electrodes with controlled Mo contents, namely PrBa_0.5_Sr_0.5_Co_1.5_Fe_0.29_Mn_0.1_Ni_0.1_Mo_0.01_O_5+δ_ (PBSCFMNM01) and PrBa_0.5_Sr_0.5_Co_1.5_Fe_0.27_Mn_0.1_Ni_0.1_Mo_0.03_O_5+δ_ (PBSCFMNM03). The unique composition of the B‐site promotes defect tolerance, high oxygen vacancy concentration, and rapid surface exchange kinetics, while at the same time ensuring exceptional phase stability under harsh environmental conditions. Furthermore, PBSCFMNM03, with its higher Mo content, exhibits enhanced oxygen‐vacancy‐related surface oxygen species, stronger hydration behavior, faster oxygen reduction reaction (ORR) kinetics, and reduced polarization resistance. When incorporated as the air electrode in R‐PCECs, PBSCFMNM03 delivers improved fuel cell performance (1.10 W cm^−2^) and enhanced electrolysis performance (1.88 A cm^−2^ at 1.3 V) at 650°C, with stable long‐term reversible operation and superior performance below 550°C. These results identify PBSCFMNM03 as an efficient air electrode for R‐PCECs and establish a multi‐element micro‐doping strategy as valuable guidance for designing air electrodes for R‐PCECs.

## Graphical abstract

Mo‐tuned multi‐element micro‐doping regulates redox chemistry, oxygen nonstoichiometry, hydration, and interfacial kinetics in PBSCF‐derived air electrodes for reversible protonic ceramic electrochemical cells. The cell with optimized PBSCFMNM03 air electrode delivers 1.10 W cm^−2^ in fuel cell mode and 1.88 A cm^−2^ at 1.3 V in electrolysis mode at 650 °C, while retaining competitive performance at 500–550 °C and 240 h reversible cycling under humid conditions.

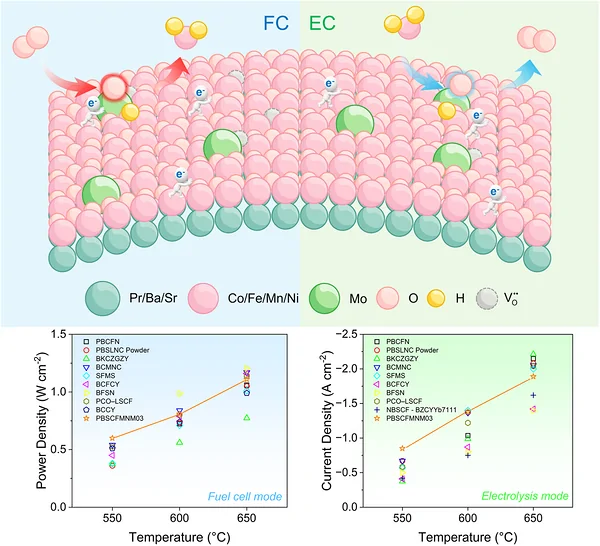

## 1 Introduction

Reversible protonic ceramic electrochemical cells (R‐PCECs) are attracting increasing interest as integrated devices for sustainable energy conversion because a single R‐PCEC can both generate electricity from chemical fuels and convert surplus electricity into hydrogen or other chemical products [1, 2, 3]. This bidirectional functionality is particularly valuable for renewable‐energy‐dominated power systems, where temporal mismatch between intermittent generation and demand necessitates efficient long‐duration energy storage. In electrolysis mode, R‐PCECs store renewable electricity in the form of hydrogen or value‐added chemicals; in fuel cell mode, they recover this stored chemical energy as dispatchable electricity [4]. Accordingly, R‐PCECs are increasingly viewed not only as hydrogen technologies, but also as flexible sector‐coupling devices linking electricity, fuels, and chemical manufacturing.

Despite these advantages, the air electrode remains a major performance and durability bottleneck in R‐PCECs. Under practical reversible operation, the same air electrode must catalyze the oxygen reduction reaction (ORR) in fuel cell mode and the water oxidation/oxygen evolution process in electrolysis mode, all under oxidizing and often steam‐rich conditions. Current advanced air electrodes include La_0.6_Sr_0.4_Co_0.2_Fe_0.8_O_3‐δ_ (LSCF) [5], Ba_0.5_Sr_0.5_Co_0.8_Fe_0.2_O_3‐δ_ (BSCF) [6, 7], BaCo_0.4_Fe_0.4_Zr_0.1_Y_0.1_O_3‐δ_ (BCFZY) [8], and PrBa_0.5_Sr_0.5_Co_1.5_Fe_0.5_O_5+δ_ (PBSCF) [2, 9, 10, 11]. Among these air electrode materials, PBSCF has emerged as a benchmark layered double perovskite because of its high mixed conductivity, proton conductivity when applied in protonic ceramic fuel cells (PCFCs), and strong reversible cell activity [10]. However, Co‐ and Sr‐containing perovskites, including PBSCF‐type electrodes, still exhibit issues with surface Sr segregation and chemical instability in CO_2_‐ and H_2_O‐containing atmospheres, which compromises long‐term durability under reversible operation [12, 13, 14, 15].

To address the activity‐stability trade‐off of perovskite air electrodes, multication and entropy‐engineering concepts have recently been explored for PCECs [16, 17, 18, 19]. High‐entropy perovskite oxides (HEPOs) generally contain several principal cations, often at comparable fractions, within a common crystallographic sublattice. Their large ideal configurational entropy can contribute to phase stabilization while expanding the compositional space available for regulating local lattice distortion, oxygen nonstoichiometry, hydration behavior, transport properties, and thermal expansion [20, 21, 22]. However, for perovskites containing multiple crystallographically distinct cation sublattices, the configurational entropy should be evaluated on a site‐resolved basis. A large nominal value obtained by summing the A‐ and B‐sublattice contributions does not necessarily demonstrate that either sublattice itself is in a high‐entropy state. Accordingly, high‐entropy perovskites are considered here as a broader materials‐design framework rather than as a classification for every multication derivative.

Recent computational and experimental work further indicates that increasing cation diversity can render the average lattice more cubic‐like while simultaneously increasing local octahedral distortion, and that the distribution of oxygen vacancy formation energies is a critical descriptor for oxygen nonstoichiometry [23]. For protonic ceramic cells, such structural robustness and defect tunability are highly desirable because the air electrode must integrate fast electronic transport with efficient oxygen ion/proton transport and rapid surface exchange under steam‐rich conditions [24].

The potential of high‐entropy and compositionally complex air electrodes for R‐PCECs has been demonstrated in several recent studies. Liu et al. reported an A‐site high‐entropy perovskite, Pr_1/6_La_1/6_Nd_1/6_Ba_1/6_Sr_1/6_Ca_1/6_CoO_3‐δ_ (PLNBSCC), as a bifunctional R‐PCEC air electrode with excellent structural stability and oxygen catalytic activity, delivering 1.21 W cm^−2^ in fuel cell mode and ‐1.95 A cm^−2^ at 1.3 V in electrolysis mode at 600°C [17]. He et al. subsequently demonstrated a high‐entropy Ce_0.2_Ba_0.2_Sr_0.2_La_0.2_Ca_0.2_CoO_3‐δ_ (CBSLCC)‐derived composite air electrode that spontaneously self‐assembles into a three‐phase electrocatalyst, reaching 1.66 W cm^−2^ and −1.76 A cm^−2^ at 1.3 V at 600°C, together with extended stability in fuel cell mode, electrolysis mode, and reversible cycling tests [25]. More recently, Kim et al. developed high‐entropy Pr_0.2_Ba_0.2_Sr_0.2_La_0.2_Nd_0.2_CoO_3‐δ_ (PBSLNC) nanofiber air electrodes that combine defect tolerance, abundant oxygen vacancies, rapid surface exchange, and exceptional phase stability, enabling 1.4 W cm^−2^ and 3.98 A cm^−2^ at 1.3 V at 650°C with stability beyond 1000 h [24]. In parallel, Oh et al. reported a high‐entropy double perovskite Pr_0.2_La_0.2_Nd_0.2_Na_0.2_Ca_0.2_Ba_0.5_Sr_0.5_Co_1.5_Fe_0.5_O_5+δ_ (PLNNCBSCF) that achieved 1.77 W cm^−2^ in fuel cell mode and 4.42 A cm^−2^ at 1.3 V in electrolysis mode at 650°C, while Sun et al. showed that entropy engineering in layered double perovskites can enhance proton uptake, oxygen vacancy concentration, and thermal stability, leading to a low polarization resistance of 0.064 Ω cm^2^ and 1.74 A cm^−2^ at 1.3 V in electrolysis mode [26]. Collectively, these studies demonstrate the potential of multication and entropy‐based materials design for regulating the activity, hydration behavior, and stability of R‐PCEC air electrodes. However, most reported systems rely on multiple principal A‐site cations, strongly multicomponent compositions, or entropy‐assisted phase reconstruction. By contrast, cobalt‐dominated PBSCF derivatives containing several minor B‐site modifiers are more appropriately distinguished from high‐entropy perovskites unless a site‐specific high‐entropy state is independently demonstrated.

Beyond conventional high‐entropy engineering, the broader principle that increased cation diversity can diversify local coordination environments has also inspired a conceptually distinct multi‐element micro‐doping strategy. Recently, Wang et al. developed a BaCoO_3‐δ_‐derived perovskite in which a total of 20% of the B sites was distributed among six dopant elements, namely Zr, Ti, Zn, In, Cu, and Mo, with the primary objective of enhancing ionic dispersion rather than maximizing configurational entropy [27]. This strategy differs from standard high‐entropy design, which typically incorporates five or more principal cations in near‐equal proportions within a common crystallographic sublattice. Instead, multi‐element micro‐doping distributes a limited total dopant concentration among several minor cations to generate spatially distributed perturbations in local metal‐oxygen bonding, cation‐valence chemistry, lattice strain, and defect energetics while preserving the dominant catalytically active host framework. It is also distinct from conventional single‐dopant modification, in which the compositional and electronic perturbation is concentrated in a single dopant species.

Inspired by the multication‐diversity concept underlying high‐entropy materials, but without seeking to maximize configurational entropy, we adopt the multi‐element micro‐doping strategy to regulate the B‐site of PBSCF. Two Mo‐tuned compositions, PrBa_0.5_Sr_0.5_Co_1.5_Fe_0.29_Mn_0.1_Ni_0.1_Mo_0.01_O_5+δ_ (PBSCFMNM01) and PrBa_0.5_Sr_0.5_Co_1.5_Fe_0.27_Mn_0.1_Ni_0.1_Mo_0.03_O_5+δ_ (PBSCFMNM03), are investigated. Relative to the parent PBSCF composition, Mn, Ni, and Mo together occupy 10.5% and 11.5% of the total B sites in PBSCFMNM01 and PBSCFMNM03, respectively. Specifically, Mn and Ni are each maintained at 5% of the B‐sites, whereas the Mo fraction is increased from 0.5% to 1.5%, with a corresponding decrease in the Fe fraction. This design distributes the compositional perturbation among three minor modifiers while retaining the Co‐rich PBSCF framework, in which Co accounts for 75% of the B‐site fraction.

Under the ideal additive cation‐mixing model adopted in this study, the nominal summed configurational entropies are 1.86*R* for PBSCFMNM01 and 1.89*R* for PBSCFMNM03. However, their corresponding B‐site configurational entropies are only 0.82*R* and 0.85*R*, respectively. Because the summed values contain a substantial common A‐site contribution and the site‐specific B‐site values remain below the conventional 1.5*R* criterion, the present materials are described as multi‐element micro‐doped PBSCF‐derived perovskites rather than B‐site HEPOs. Accordingly, the comparison between PBSCFMNM01 and PBSCFMNM03 is interpreted primarily as a Mo‐concentration‐dependent compositional effect within a fixed Mn/Ni‐containing framework.

The selection of the PBSCFMNM compositions was guided by previous compositional screening and by reported effects of Mn and Ni in Pr‐Ba‐Co‐based air electrodes. In our earlier investigation of PrBa_0.5_Sr_0.5_Co_1.5_Fe_0.5‐x_Mo_x_O_5+δ_, the compositions with nominal x = 0.01 and 0.03 retained the PBSCF‐derived perovskite phase, whereas x = 0.05 produced a detectable BaMoO_4_ secondary phase [15]. Therefore, x = 0.01 and 0.03 were selected as dilute and upper phase‐pure Mo levels, respectively, rather than as an exhaustive optimization of Mo content. Mn and Ni were introduced at fixed nominal contents of 0.10 each, corresponding to 5% of the total B‐site population per element. Mn was selected because its accessible Mn^3+^/Mn^4+^ redox states can provide additional charge compensation pathways and regulate oxygen nonstoichiometry and local metal‐oxygen coordination. Previous investigations of Pr‐Ba‐Co‐based perovskites have shown that moderate Mn incorporation can reduce thermal expansion and improve electrode activity, whereas excessive Mn substitution may decrease performance [28]. Ni was selected because Ni substitution can modify B─O bond strength and oxygen vacancy formation thermodynamics; appropriate Ni incorporation has also been reported to decrease electrode polarization resistance and thermal expansion [29]. The relatively low and equal Mn and Ni contents were therefore chosen to disrupt the local redox and defect chemistry while preserving the Co‐rich mixed conducting framework. Because Mn and Ni are held constant in both compositions, the present comparison is intended specifically to determine the influence of increasing the Mo content from x = 0.01 to x = 0.03, rather than to isolate the independent effects of Mn or Ni.

The two compositions are systematically evaluated in terms of phase structure, compositional homogeneity, oxygen nonstoichiometry, hydration response, surface oxygen chemistry, electrical transport, oxygen reduction kinetics, thermomechanical properties, and stability under steam‐containing atmospheres. R‐PCECs incorporating the PBSCFMNM_x_ air electrodes are subsequently examined in both fuel cell and electrolysis modes to correlate the controlled increase in Mo content with electrode reaction kinetics and reversible electrochemical performance. This approach evaluates whether low‐level multi‐element B‐site regulation can preserve the intrinsic activity of the PBSCF backbone while modifying the redox, defect, and hydration characteristics relevant to intermediate temperature R‐PCEC operation.

## 2 Results and Discussion

### 2.1 Structural and Morphological Analysis of PBSCFMNM_x_

Detailed structural and compositional analyses were conducted on samples PBSCFMNM01 and PBSCFMNM03 to confirm their phase integrity, structural characteristics, and compositional homogeneity. Figure 1a schematically illustrates the sol–gel synthesis route used to prepare the B‐site compositionally regulated PBSCFMNM_x_. This route follows a process using citric acid and ethylene glycol, followed by heating and calcination, which is appropriate for dispersing the multiple B‐site cations before crystallization. The porous powder morphology shown schematically after the calcination step in Figure 1a is consistent with the intended use as an air electrode, and it is a phase‐pure oxide with dispersed transition‐metal sites which can simultaneously possess the capabilities for surface oxygen exchange, bulk oxygen transport, and electronic conduction. The phase structures of PBSCF, PBSCFMNM01, and PBSCFMNM03 were first examined by XRD, as shown in Figure 1b. All samples exhibit sharp and well‐defined diffraction peaks that can be indexed to PDF#00‐053‐0131 [17, 30, 31]. PDF#00‐053–0131 is used as a reference for the parent PrBaCo_2_O_5+δ_ structural family, whereas the structural assignment of the Mn/Ni/Mo‐substituted compositions is evaluated through comparative Rietveld refinements. No additional reflections corresponding to impurity oxides or segregated Mn─, Ni─, or Mo‐containing secondary phases are detected within the instrumental resolution, indicating that the introduced Mn, Ni, and Mo species are successfully accommodated in the PBSCF‐derived lattice. This is consistent with prior literature showing that PBSCF‐type materials crystallize as tetragonal layered double perovskites related to a P4/mmm space group [9], and that this group is capable of tolerating substantial B‐site compositional tuning while preserving the layered backbone.

**FIGURE 1:**
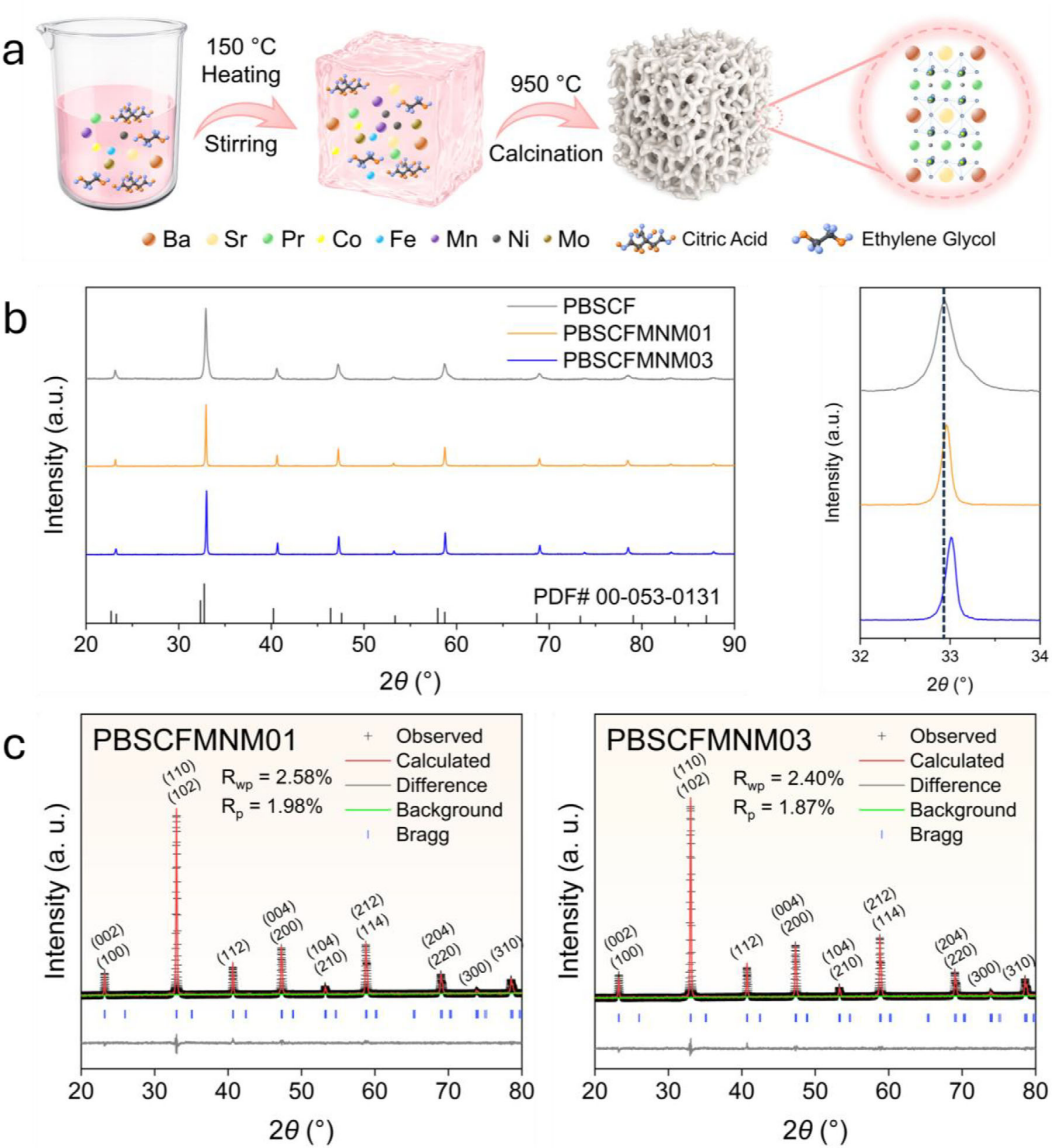
Synthesis and crystallographic characterization of PBSCFMNM_x_. (a) Schematic diagram of the sol–gel synthesis method. (b) x‐ray diffraction (XRD) patterns of PBSCFMNM_x_. (c) Rietveld refinement results of XRD patterns of PBSCFMNM_x_.

Moreover, the enlarged XRD patterns around the strongest principal (110) peak near 33° in Figure 1a reveal a slight composition‐dependent peak shift for the doped samples. The doped compositions PBSCFMNM01 and PBSCFMNM03 show a slight but systematic shift of the main peak toward a higher diffraction angle, with PBSCFMNM03 appearing marginally more shifted than PBSCFMNM01. By Bragg's law [32], this directly implies a small change in the average interplanar spacing after multication substitution. Since no new diffraction peaks emerge and the peak family itself is preserved, the shift should be interpreted as a subtle lattice perturbation rather than a phase transition. This shift is attributed to the partial substitution of cations with smaller ionic radii at the B‐site of Fe by the multi‐element micro‐doping strategy. In detail, the smaller ionic radii of Mn^4+^ (0.53 Å), Ni^2+^ (0.69 Å), and Mo^6+^ (0.59 Å) compared to Fe^3+^ (0.78 Å)/Fe^4+^ (0.59 Å) [33] result in a reduction in the average B‐site ionic radii and, therefore, a reduction in the lattice dimension. Rietveld refinements were then performed to further support the phase assignment and quantify the small structural response to the increased Mo content. In Figure 1c, both patterns are fitted by a tetragonal P4/mmm model, and the low residuals indicate a satisfactory match between calculated and observed profiles. The refinement quality is slightly improved for PBSCFMNM03, with R_wp_ and R_p_ values of approximately 2.40% and 1.87%, compared with approximately 2.58% and 1.98% for PBSCFMNM01. The lattice parameters from Table S1 show a slight contraction from a = b = 3.860 Å and c = 7.720 Å for PBSCFMNM01 to a = b = 3.858 Å and c = 7.716 Å for PBSCFMNM03, with the unit cell volume decreasing from 115.025 to 114.864 Å^3^. The lattice contraction is small but systematic across all lattice dimensions. This trend is consistent with a modified average B‐site environment caused by the (Mn/Ni/Mo)‐for‐Fe adjustment (specifically Mo) and associated charge compensation. More importantly, the lattice contraction occurs without loss of the layered symmetry, showing that PBSCFMNM03 preserves the crystallographic foundation required for oxygen‐defect‐mediated transport.

Chemical compatibility with adjacent electrolyte materials is another prerequisite for applying these perovskites as air electrodes for R‐PCECs. Figure S1 compares XRD patterns of PBSCFMNM03 mixed with BaZr_0.1_Ce_0.7_Y_0.1_Yb_0.1_O_3‐δ_ (BZCYYb) and Gd_0.1_Ce_0.9_O_2‐δ_ (GDC) after calcination at 1100°C for 20 h. The diffraction features of the electrode and electrolyte components indicate that PBSCFMNM03 does not undergo an obvious deleterious solid‐state reaction with either BZCYYb or GDC under the tested thermal conditions. This observation is particularly relevant because later electrochemical analyses use BZCYYb‐ and GDC‐supported symmetric cells to separate electrode kinetics from full cell behavior. The compatibility data also reduce the likelihood that the improved impedance response of PBSCFMNM03 originates from secondary reaction products at the electrode‐electrolyte interface. Instead, the structural and compatibility evidence together indicates that PBSCFMNM03 is a chemically viable air electrode phase whose enhanced performance can be examined within the same perovskite structural family as PBSCFMNM01.

Transmission electron microscopy (TEM)‐based morphological characterization was further performed to verify whether the Mn/Ni/Mo co‐substituted PBSCF lattice retains local crystallinity and nanoscale compositional uniformity. As illustrated in Figure 2a,b, the high‐resolution TEM (HRTEM) images display continuous and well‐resolved lattice fringes over the selected regions, and the FFT patterns present distinct diffraction spots rather than diffuse rings, confirming the locally well‐crystallized nature of PBSCFMNM01 and PBSCFMNM03. Notably, the measured interplanar spacing decreases slightly from 0.275 nm for PBSCFMNM01 to 0.272 nm for PBSCFMNM03. This variation agrees well with the subtle peak shift observed in Figure 1, indicating that Mo enrichment introduces a measurable local lattice contraction. Such a slight contraction is likely related to the collective modification of B─O bonding and oxygen defect equilibrium caused by replacing part of Fe with Mo in the multication Co/Fe/Mn/Ni/Mo‐O sublattice.

**FIGURE 2:**
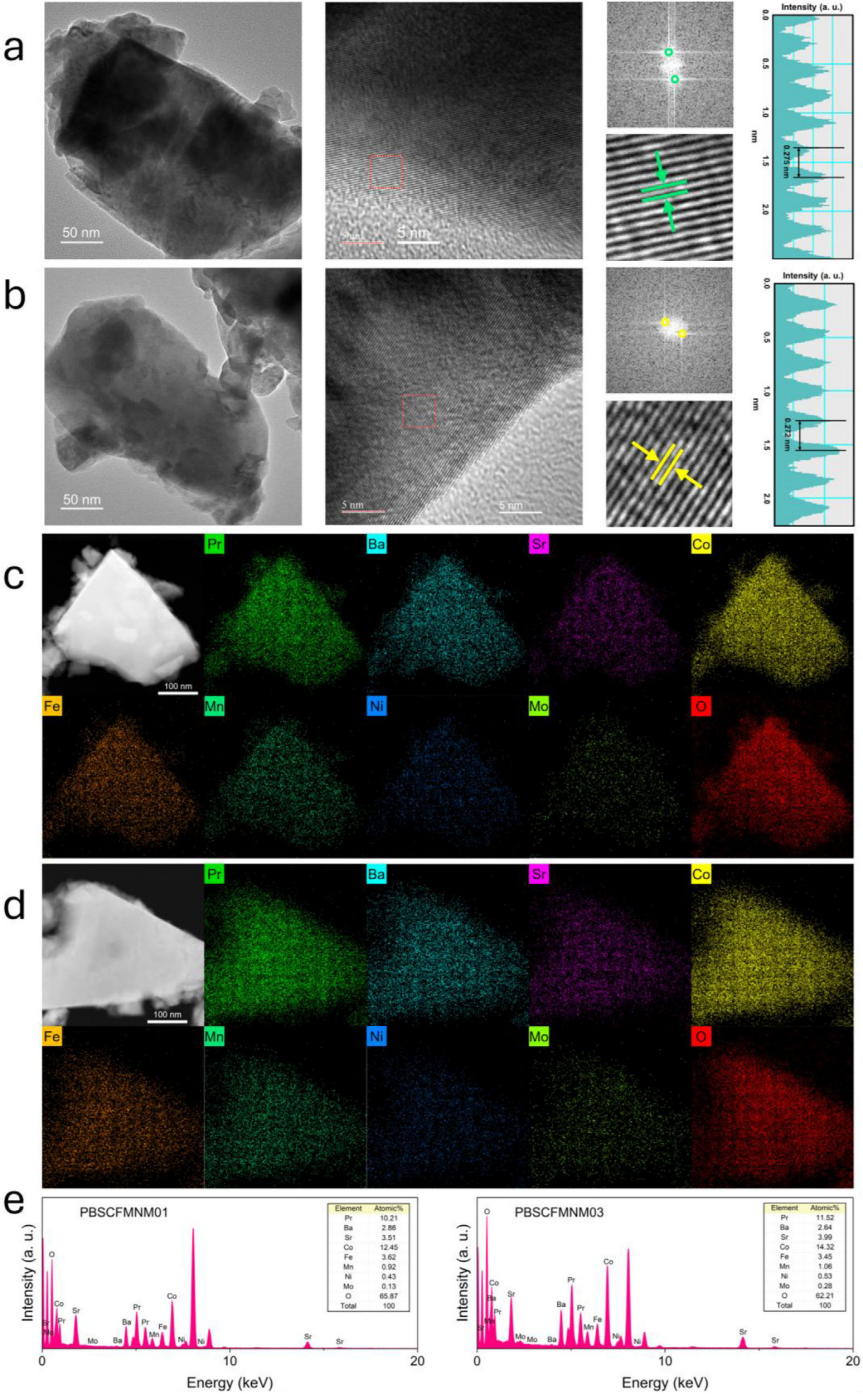
Transmission electron microscopy (TEM) analysis and compositional characterization of PBSCFMNM_x_. High‐resolution transmission electron microscopy (HRTEM), fast Fourier transform (FFT), and d‐spacing for (a) PBSCFMNM01 and (b) PBSCFMNM03. Scanning transmission electron microscopy with energy‐dispersive spectroscopy (STEM‐EDS) elemental mapping of (c) PBSCFMNM01 and (d) PBSCFMNM03. (e) EDS spectra of PBSCFMNM01 (left) and PBSCFMNM03 (right).

To further provide direct evidence for the spatial distribution of the constituent cations, STEM‐EDS elemental mappings were performed. In Figure 2c, the elemental maps of PBSCFMNM01 show that Pr, Ba, Sr, Co, Fe, Mn, Ni, Mo, and O are uniformly distributed throughout the selected particle. In particular, the intentionally introduced Mn, Ni, and Mo species are co‐localized with the parent PBSCF elements, without obvious element‐rich clusters or depleted regions. Although the Mo signal is relatively weak because of its low nominal concentration, it follows the particle contour rather than forming isolated hotspots, supporting successful incorporation of Mo into the oxide matrix within the spatial resolution of STEM‐EDS. Figure 2d confirms that PBSCFMNM03 also maintains homogeneous elemental distribution after increasing the Mo content. All cationic elements and oxygen remain broadly co‐distributed across the particle, and no Mo‐rich secondary domain is detected. Compared with PBSCFMNM01, the Mo map of PBSCFMNM03 appears more populated, which is consistent with the higher nominal Mo concentration. This observation is important because it demonstrates that the PBSCF‐derived lattice can accommodate the increased Mo amount without inducing detectable nanoscale segregation. Therefore, the higher Mo sample preserves the structural and compositional continuity required for efficient mixed ionic‐electronic‐protonic transport and surface oxygen exchange.

The EDS spectra in Figure 2e further support the compositional distinction between the two samples. The spectra show signals corresponding to the A‐site species Pr, Ba, and Sr; the dominant B‐site Co and Fe; the additional B‐site Mn, Ni, and Mo; and oxygen, while the most relevant comparison is the increase in Mo content from 0.13 to 0.28 at.%, accompanied by a slight decrease in Fe content from 3.62 to 3.45 at.%, which is directionally consistent with the designed Fe‐site replacement from PBSCFMNM01 to PBSCFMNM03. Since TEM‐EDS is semi‐quantitative, especially for oxygen and low‐concentration dopants, these values should not be overinterpreted as exact bulk stoichiometries. Nevertheless, they clearly confirm the presence of all designed elements and verify the relative enrichment of Mo in PBSCFMNM03. The agreement between STEM‐EDS maps and EDS spectra strengthens the interpretation that PBSCFMNM01 and PBSCFMNM03 are not merely mixtures of simpler oxides, but multication perovskites with distributed cation populations. This distinction matters mechanistically because multication perovskites are expected to contain local variations in bond strength, cation valence, and oxygen coordination. Such local disorder can broaden oxygen migration pathways and tune the energetic landscape of oxygen vacancy formation, provided that the global structure remains coherent.

### 2.2 Oxygen Vacancy and Physicochemical Properties of PBSCFMNM_x_

Following the phase‐pure crystallization and homogeneous Mn/Ni/Mo incorporation confirmed in Figures 1 and 2, the oxygen vacancy and physicochemical properties of the PBSCFMNM_x_ were evaluated using TGA, O_2_‐TPD, XPS, and thermal expansion analysis. Figure 3a presents TGA curves of PBSCFMNM_x_ measured in dry N_2_ from 25°C to 1000°C. Both materials undergo gradual weight loss with increasing temperature, which is typical of oxygen release from oxygen‐deficient perovskite oxides at elevated temperatures. In the low‐temperature region (< 400°C), the slight weight decrease is mainly attributed to the removal of physically adsorbed oxygen and weakly bound surface oxygen species. PBSCFMNM03 shows a more pronounced initial weight loss than PBSCFMNM01, suggesting that higher Mo incorporation induces a larger amount of surface‐adsorbed oxygen species, likely associated with a higher surface defect concentration or more oxygen‐vacancy‐related adsorption sites. At higher temperatures, the mass loss becomes dominated by lattice oxygen species released from the perovskite framework. This process is commonly related to the formation of oxygen vacancies and the corresponding partial reduction of multivalent B‐site cations such as Co, Fe, Mn, Ni, and Mo to balance the formed oxygen vacancies [34]. PBSCFMNM03 exhibits a larger oxygen‐related weight loss of approximately 1.173%, compared with 0.817% for PBSCFMNM01. This indicates that PBSCFMNM03 contains more labile lattice oxygen and has a higher oxygen nonstoichiometry, reflecting enhanced oxygen vacancy formation and greater lattice oxygen mobility, which is beneficial for oxygen surface exchange and oxygen ion transport [15, 35].

**FIGURE 3:**
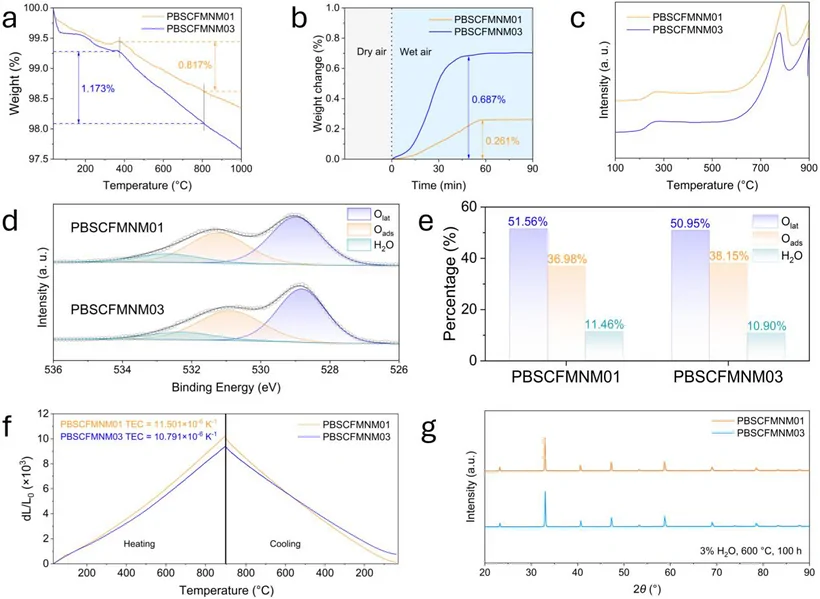
Characterization of the oxygen‐vacancy‐related properties, hydration behavior, thermomechanical properties, and steam stability of PBSCFMNM_x_. (a) Thermogravimetric analysis (TGA) curves. (b) TG relaxation curves. (c) Oxygen temperature‐programmed desorption (O_2_‐TPD) results. (d) x‐ray photoelectron spectroscopy (XPS) spectra of O 1*s*. (e) Proportion of O 1*s* species. (f) Dilatometry test results. (g) XRD patterns of steam‐treated samples.

Consistently, the wet‐air relaxation experiment in Figure 3b further links the Mo adjustment to hydration‐related behavior. After switching from dry air to wet air, both samples show a positive TG relaxation response, indicating mass uptake from water‐related hydration. However, PBSCFMNM03 exhibits a much larger and faster weight increase than PBSCFMNM01 (0.687% vs. 0.261%), demonstrating that the higher Mo composition possesses markedly enhanced hydration ability. In proton‐conducting cell environments, a larger wet‐air mass uptake is commonly associated with stronger interaction with water vapor, which can include hydration of oxygen vacancies and incorporation of protonic defects near the electrode surface [36].

(1) $$H_{2}O+O_{O}^{\times }+V_{O}^{••}\to 2{\mathrm{OH}}_{O}^{•}$$

The higher response of PBSCFMNM03 therefore suggests that its oxygen vacancies are not only more abundant but also more available for hydration‐related surface chemistry. This feature is important for R‐PCECs because the air electrode must support ORR during fuel cell operation and oxygen evolution reaction (OER) or steam‐related electrochemical processes during electrolysis mode operation. The wet‐air relaxation behavior thus complements the TGA evidence by showing that PBSCFMNM03 is more responsive to the humid oxidizing atmosphere relevant to R‐PCEC operation.

Figure 3c further supports this interpretation, as the O_2_‐TPD profiles indicate modified oxygen desorption behavior after increasing Mo content. The low‐temperature *α*‐oxygen peak at around 200–300°C is attributed to surface‐adsorbed oxygen species, while the high‐temperature *β*‐oxygen peak above approximately 700°C corresponds mainly to the release of lattice oxygen from the perovskite structure [31, 37]. This desorption is related to bulk oxygen vacancy formation and the reducibility of multivalent B‐site cations. The strong *β*‐oxygen peak signals of PBSCFMNM03 suggest more readily activated lattice oxygen and stronger oxygen exchange behavior. Although O_2_‐TPD peak assignment can be complex in multication perovskites, the comparison supports the same general conclusion drawn from TGA: increasing the Mo content from 0.01 to 0.03 alters oxygen nonstoichiometry and oxygen exchange characteristics without destroying the layered phase. In air electrodes, more labile oxygen species and accessible vacancies can accelerate the steps of oxygen adsorption, dissociation, charge transfer, and incorporation. The O_2_‐TPD data therefore provide a bridge between structural chemistry and electrochemical behavior, suggesting why PBSCFMNM03 should exhibit lower polarization resistance in later symmetric cell and full cell tests.

In parallel with these bulk defect‐sensitive results, Figure 3d,e reveal that the surface oxygen chemistry is also altered by Mo enrichment. XPS analysis of the O 1*s* spectra directly probes the surface oxygen environment, which is particularly relevant because the ORR begins at or near the electrode surface. Figure 3d shows deconvoluted O 1*s* spectra assigned to lattice oxygen (O_lat_), adsorbed oxygen species (O_ads_), and surface H_2_O‐related components, while the quantitative results in Figure 3e and Table S2 show that PBSCFMNM03 contains a slightly higher fraction of O_ads_ than PBSCFMNM01 (38.15% vs. 36.98%), together with a slightly lower lattice oxygen fraction (50.95% vs. 51.56%). As a result, the O_ads_/O_lat_ ratio increases from 0.72 for PBSCFMNM01 to 0.75 for PBSCFMNM03. Because adsorbed oxygen species are often associated with surface oxygen vacancies and active oxygen exchange sites [15, 38, 39], this higher O_ads_/O_lat_ ratio provides direct surface‐sensitive support for the claim that PBSCFMNM03 possesses a higher oxygen‐vacancy‐related active‐site population. Although XPS fitting is surface‐sensitive and should be interpreted semi‐quantitatively, this trend agrees well with Figure 3a–c, indicating that a modest increase in oxygen‐vacancy‐related surface oxygen species and the higher Mo concentration increase the availability of surface defect sites capable of stabilizing reactive oxygen species.

The XPS survey spectra in Figure S2 confirm that both PBSCFMNM01 and PBSCFMNM03 contain the expected cation and oxygen signals, while the high‐resolution spectra and fitted values in Figures S3–S6 and Tables S3–S6 reveal how charge compensation changes across the multication lattice. Figure S3 and Table S3 show that the Co^3+^ fraction increases from 45.82 at.% in PBSCFMNM01 to 50.63 at.% in PBSCFMNM03, while the Co^4+^ fraction decreases from 54.18 to 49.37 at.%. Figure S4 and Table S4 illustrate a smaller change in Fe, with Fe^4+^ increasing from 47.66 to 48.32 at.% and Fe^3+^ decreasing from 52.34 to 51.68 at.%. Figure S5 and Table S5 demonstrate that the Pr^4+^ fraction increases from 62.26 to 68.70 at.% in PBSCFMNM03. Figure S6 and Table S6 show that the Mn^4+^ fraction increases from 18.98 to 20.44 at.%. These coupled valence changes indicate that the system compensates for the higher Mo content through a redistribution of oxidation states among several cations rather than through a single isolated redox couple. The valence redistribution is important because oxygen vacancies in complex multication perovskites are controlled by the balance among high‐valence dopants, transition‐metal oxidation states, and oxygen nonstoichiometry. The decrease in Co^4+^ fraction in PBSCFMNM03 suggests that part of the charge adjustment reduces the average cobalt valence, while the increases in Pr^4+^, Fe^4+,^ and Mn^4+^ indicate that charge is also redistributed across A‐site and B‐site species. Such a multichannel compensation mechanism is plausible in a multication lattice, where several redox‐active elements coexist, and local bonding environments vary. The net surface signature, however, is clear: PBSCFMNM03 shows a higher O_ads_/O_lat_ ratio and stronger wet‐air mass uptake. These results indicate that the higher Mo level favors a defect environment with more accessible oxygen‐vacancy‐related sites. The XPS fitting also helps bound the mechanism. The improvement is not simply due to a higher content of one reducible cation; instead, it arises from the collective adjustment of a multication lattice in response to a small increase in high‐valence Mo.

Figure S7 and Table S7 address Sr surface chemistry, which is an important degradation concern for perovskite air electrodes. The fitted Sr 3*d* data show a surface‐Sr/lattice‐Sr ratio of 0.689 for PBSCFMNM01 and 0.678 for PBSCFMNM03. The slightly lower ratio for PBSCFMNM03 indicates that increasing Mo to 0.03 does not promote stronger Sr surface segregation in the measured samples; instead, the fitted surface Sr contribution is marginally reduced. This observation is helpful because enhanced oxygen vacancy concentration can sometimes be accompanied by cation segregation or surface reconstruction, which would compromise long‐term electrode activity. Here, the XPS data suggest that PBSCFMNM03 improves the oxygen‐vacancy‐related surface chemistry without an obvious penalty in Sr enrichment. This balance is desirable for R‐PCECs, where the air electrode is exposed to oxidizing, humid, and polarized conditions that can accelerate surface instability.

Importantly, the enhanced defect activity of PBSCFMNM03 does not sacrifice thermomechanical or chemical stability. Thermomechanical compatibility of PBSCFMNM_x_ was evaluated by dilatometry in Figure 3f. Both PBSCFMNM01 and PBSCFMNM03 show smooth expansion and contraction behavior during heating and cooling, with no abrupt anomaly that would indicate a major phase transition in the tested temperature window. The calculated thermal expansion coefficients (TECs) are low for cobaltite‐based air electrodes, approximately 11.501 × 10^−6^ K^−1^ for PBSCFMNM01 and 10.791 × 10^−6^ K^−1^ for PBSCFMNM03. In Co‐rich perovskites, thermal expansion contains both normal lattice expansion and a chemical expansion associated with oxygen loss, transition metal reduction, and Co spin‐state changes [40]. Partial substitution of Fe by Mn, Ni, and Mo modifies the B─O bonding environment and may reduce the dimensional response associated with these thermally induced redox and defect changes. In particular, the comparatively rigid Mo‐containing B─O linkages and the compact multication framework may constrain local defect‐induced strain. The slight unit‐cell contraction with increasing Mo content is consistent with this interpretation. Nevertheless, because the individual lattice, redox, and spin‐state contributions were not independently resolved, the relatively low TEC is attributed cautiously to the combined effects of strengthened B─O interactions and reduced chemical expansion rather than to a single compositional or entropy‐related factor. The slightly lower TEC of PBSCFMNM03 is favorable for integration with proton‐conducting electrolytes because thermal mismatch can generate mechanical stress during cell fabrication and thermal cycling. Figure S8 compares these values with several PrBaCo_2_O_5+δ_‐based air electrodes reported in the supporting references. PBSCFMNM01 and PBSCFMNM03 occupy the lowest‐TEC region of this comparison, indicating that multi‐element micro‐doping engineering and the small Mo increase do not create the large thermal expansion often associated with highly reducible cobalt‐rich perovskites but instead reduce the thermal expansion substantially.

Steam stability of PBSCFMNM_x_ was examined by XRD after exposure to 3% H_2_O at 600°C for 100 h, as shown in Figure 3g. The diffraction patterns of both PBSCFMNM01 and PBSCFMNM03 retain the characteristic perovskite reflections after the steam treatment, with no obvious new impurity peaks visible in the displayed range. This result is critical because R‐PCECs operate in humid atmospheres, and air electrodes can undergo hydration‐induced decomposition, cation segregation, or phase transformation. The stability of the XRD patterns indicates that both air electrodes tolerate the tested wet‐air condition, while the defect and hydration data show that PBSCFMNM03 remains more responsive to oxygen and water‐related chemistry. The combination of steam stability and enhanced oxygen vacancy signatures is therefore central to the material design. PBSCFMNM03 does not achieve higher activity by becoming structurally fragile; instead, it retains the perovskite framework while increasing the population or accessibility of reactive oxygen species.

### 2.3 Oxygen Reduction Kinetics and Electrode Transport Behavior

Electrochemical impedance spectroscopy (EIS) was conducted to investigate the ORR activities of PBSCFMNM_x_ electrodes on electrolyte‐supported symmetric cells. Figure 4a compares the Nyquist plots of PBSCFMNM01 and PBSCFMNM03 on a BZCYYb‐supported symmetric cell at 550°C in 3% H_2_O wet air, together with the corresponding DRT analysis. Figures S9 and S10 expand the comparison of EIS and DRT results of PBSCFMNM_x_ electrodes in a BZCYYb symmetric cell across 500–650°C. The impedance arcs are composed of multiple contributions, as expected for porous mixed‐conducting air electrodes where gas diffusion, surface oxygen exchange, charge transfer, and oxygen incorporation can overlap. For both electrodes, the impedance arcs shrink as temperature increases, reflecting faster thermally activated electrode reactions. At each temperature, PBSCFMNM03 shows a lower polarization resistance (R_p_) than PBSCFMNM01, indicating that increasing the Mo content from 0.01 to 0.03 enhances the intrinsic electrode ORR kinetics under protonic ceramic cell‐relevant humid air, and the difference is especially crucial at lower operating temperatures where air electrode kinetics usually become limiting. To gain a deeper understanding of ORR kinetics, the EIS spectra of both air electrodes in BZCYYb‐supported symmetric cells were analyzed utilizing the DRT tools to deconvolute distinct electrochemical processes [41]. The DRT spectra in Figure 4a and Figure S10 further reveal that this improvement is not associated with a single isolated step, but with the suppression of multiple processes. The low‐frequency (LF), intermediate‐frequency (IF), and high‐frequency (HF) regions are generally related to gas diffusion or adsorption‐related processes within the electrode, surface exchange coupled with bulk diffusion, and interfacial charge transfer processes, respectively [35, 41, 42]. The DRT profiles demonstrate that the Mo‐enriched composition reduces contributions across the LF, IF, and HF regions, although the relative magnitude of each process changes with temperature. The broad IF response is particularly relevant because it often contains contributions from oxygen surface exchange and incorporation processes. Suppression of this region in PBSCFMNM03 is consistent with improved oxygen vacancy availability and faster oxygen exchange. This is consistent with the enhanced oxygen vacancy and adsorbed oxygen signatures observed in Figure 3.

**FIGURE 4:**
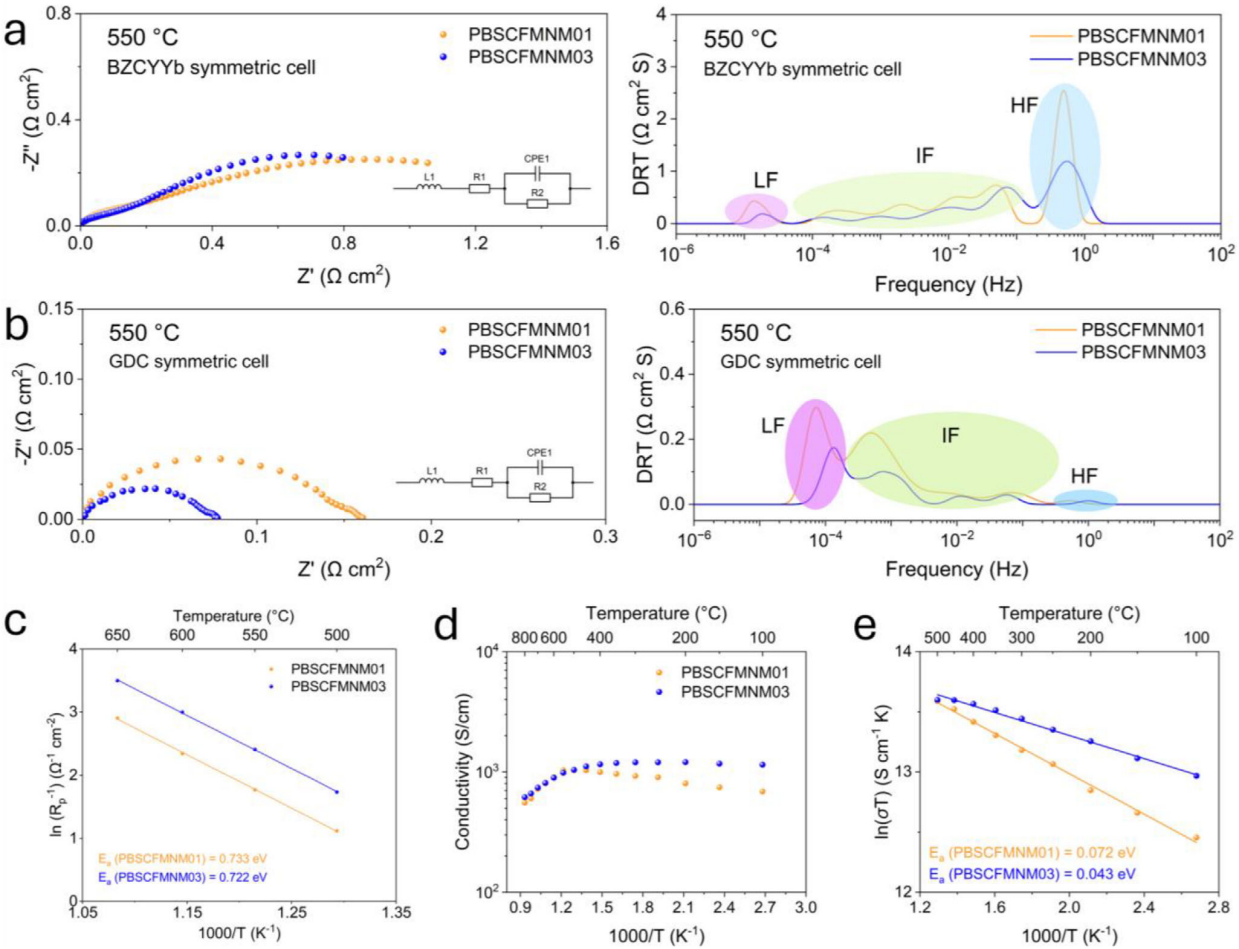
ORR activity and transport properties of PBSCFMNM01 and PBSCFMNM03 air electrodes. (a) Nyquist plots of a BZCYYb electrolyte‐supported symmetric cell with PBSCFMNM_x_ air electrodes at 550°C in 3% H_2_O wet air and distribution of relaxation times (DRT) results. (b) Nyquist plots of a GDC electrolyte‐supported symmetric cell with PBSCFMNM_x_ air electrodes at 550°C in 3% H_2_O wet air and DRT results. (c) Arrhenius plots of the R_p_ values of the symmetric cells. (d) Electrical conductivity of PBSCFMNM_x_ air electrodes. (e) Arrhenius plots of the electrical conductivity of PBSCFMNM_x_ from 100°C to 500°C.

The same trend is more evident in the GDC‐supported symmetric cells shown in Figure 4b and Figures S11, and S12. Across 500–650°C, PBSCFMNM03 exhibits a much smaller Nyquist arc than PBSCFMNM01, demonstrating lower R_p_ for ORR on the oxygen ion‐conducting electrolyte. The DRT spectra show diminished LF and IF contributions for PBSCFMNM03, suggesting that the improvement affects both surface‐related and transport‐coupled processes. This repeated trend across different electrolytes strengthens the conclusion that PBSCFMNM03 has faster ORR kinetics as an electrode material, not simply a lower interfacial resistance under one specific testing configuration. The use of both BZCYYb‐ and GDC‐supported symmetric cells for EIS tests is useful because it shows that the kinetic improvement is not restricted to a single electrolyte interface. BZCYYb is relevant to R‐PCECs, while GDC provides an oxygen ion‐conducting platform that helps assess air electrode activity under a different interfacial environment. The consistent reduction in R_p_ across both symmetric cells indicates that PBSCFMNM03 has intrinsically improved air electrode kinetics.

The Arrhenius analysis in Figure 4c quantifies the temperature dependence of R_p_. Both PBSCFMNM_x_ electrodes show thermally activated behavior. The apparent activation energy (*E_a_*) is slightly lower for PBSCFMNM03, approximately 0.722 eV, compared with approximately 0.733 eV for PBSCFMNM01. Although the difference is modest, it is consistent with the compositional change being intentionally small. The meaningful point is that the higher Mo composition decreases the R_p_ while slightly lowering the apparent kinetic barrier. This behavior agrees with the defect chemistry results: a higher density of oxygen‐vacancy‐related surface sites can reduce the energetic penalty for oxygen incorporation and charge transfer.

Electrical conductivity measurements in Figure 4d,e show that both PBSCFMNM01 and PBSCFMNM03 maintain suitable electronic transport in the intermediate‐temperature range. The conductivity remains on the order required for triple‐conducting air electrodes and varies gradually with temperature. After the conductivity reaches its maximum at around 400°C, further increases in temperature promote oxygen release from the lattice and the partial reduction of Co^4+^ to Co^3+^, which decreases the concentration of electron holes responsible for *p*‐type conduction. In addition, the oxygen vacancies generated at elevated temperature may interrupt the continuous B─O─B hopping pathway and increase lattice disorder, thereby suppressing small‐polaron transport [15]. PBSCFMNM03 displays a slightly different conductivity‐temperature relationship from PBSCFMNM01, and the Arrhenius fits in Figure 4e give a lower conduction *E_a_* for PBSCFMNM03, approximately 0.043 eV, compared with approximately 0.072 eV for PBSCFMNM01. This lower *E_a_* indicates easier charge transport over the measured temperature range of 100–500°C. However, the electrochemical advantage of PBSCFMNM03 should not be attributed only to electronic conductivity, because the most significant changes appear in the oxygen surface chemistry and R_p_ response. Rather, the conductivity data show that the Mo increase preserves adequate electronic conduction while the defect structure improves the ORR kinetics.

### 2.4 Reversible Single Cell Performance and Durability

The practical relevance of the improved air electrode kinetics was evaluated in full R‐PCECs. Figure 5a shows a cross‐sectional SEM image of an R‐PCEC cell using a PBSCFMNM03 air electrode, a dense BZCYYb electrolyte, and a porous Ni‐BZCYYb fuel electrode. The electrolyte layer appears continuous and dense, while both electrodes are porous, providing the expected architecture for gas diffusion, charge transfer, and electrochemical reaction. The PBSCFMNM03 air electrode is well attached to the electrolyte without an obvious delamination gap in the displayed cross‐section. This microstructure is critical since full cell performance depends not only on intrinsic electrode activity but also on the formation of a continuous electrode‐electrolyte interface.

**FIGURE 5:**
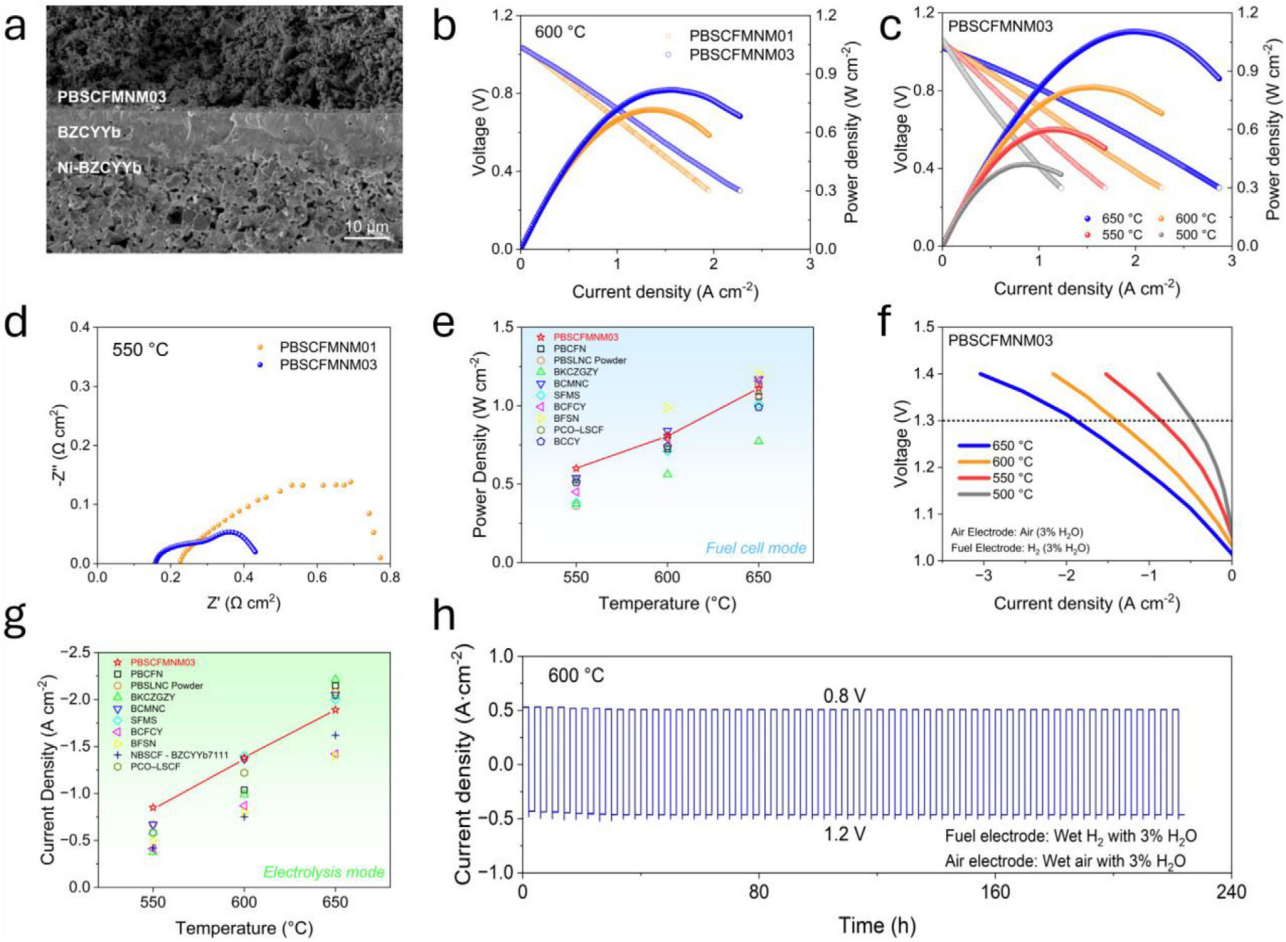
Electrochemical performance and durability of R‐PCECs with PBSCFMNM03 air electrode. (a) Cross‐sectional scanning electron microscopy (SEM) image of R‐PCEC with PBSCFMNM03 air electrode. (b) I‐V‐P curves of R‐PCEC with PBSCFMNM01 and PBSCFMNM03 air electrodes at 600°C in fuel cell mode, and (c) I‐V‐P curves of R‐PCEC with PBSCFMNM03 air electrode at various temperatures in fuel cell mode. (d) Nyquist plots of R‐PCECs with PBSCFMNM01 and PBSCFMNM03 electrodes at 550°C. (e) Peak power density (PPD) comparison of the PBSCFMNM03 R‐PCEC with other published PCECs. (f) *I–V* curves of R‐PCEC with PBSCFMNM03 air electrode at various temperatures in electrolysis mode. (g) Current density comparison of the PBSCFMNM03 cell with other R‐PCECs at 1.3 V. (h) Long‐term stability test of an R‐PCEC with PBSCFMNM03 air electrode at 600°C.

Fuel cell‐mode I‐V‐P curves illustrated in Figure 5b compare the electrochemical performance of R‐PCECs using PBSCFMNM01 and PBSCFMNM03 air electrodes at 600°C. Further, the temperature‐dependent I‐V‐P curves of the cells with PBSCFMNM03 and PBSCFMNM01 over the range of 500–650°C are shown in Figure 5c and Figure S13, respectively. The PBSCFMNM03 cell delivers higher power densities than the PBSCFMNM01 cell across the whole temperature range, with 1.10, 0.81, 0.60, and 0.42 W cm^−2^ at 650°C, 600°C, 550°C, and 500°C, respectively, compared to 0.91, 0.72, 0.54, and 0.38 W cm^−2^. The PBSCFMNM03 cell not only achieves a higher PPD but also exhibits a less severe voltage decline with increasing current density, indicating reduced total cell polarization. Since the fuel electrode and electrolyte architecture are otherwise comparable, this improvement is attributed mainly to the air electrode difference. The result is consistent with the symmetric cell measurements, where PBSCFMNM03 showed lower R_p_ on both BZCYYb and GDC‐supported cells. In other words, the faster ORR kinetics of Mo‐enriched PBSCFMNM03 observed in symmetric cell testing are successfully transferred to the full R‐PCEC configuration, confirming that the Mo‐enriched air electrode improves device‐level fuel cell mode output. The full cell impedance spectra in Figure 5d further support this interpretation. At 550°C, the PBSCFMNM03‐based cell displays a smaller polarization than the PBSCFMNM01‐based cell. This lower impedance indicates that the air electrode improvement remains active under realistic full cell operation, where gas atmospheres, electrode thickness, current collection, and electrode‐electrolyte interfaces all contribute to the measured response. Figure S14 extends the full cell Nyquist analysis across 500–650°C for PBSCFMNM01 and PBSCFMNM03. As expected, the impedance decreases with increasing temperature for both cells, but PBSCFMNM03 maintains a substantially smaller response throughout the measured range. Figure S15 combines comparative Nyquist and DRT analysis for the full cells across 500–650°C, showing reduced DRT contributions for PBSCFMNM03 in all three regions. These full‐cell EIS results show that the same ORR kinetic advantage observed in symmetric cells is retained in full reversible cells. Figure 5e compares the PBSCFMNM03 performance with other recently reported air electrodes of R‐PCECs. PBSCFMNM03 lies among the higher‐performing entries in the comparison and exhibits particularly superior performance at the relatively low temperature.

Electrolysis mode tests of the PBSCFMNM01 and PBSCFMNM03 cells were further conducted as shown in Figure 5f and Figure S16, where both cells were tested from 650 to 500°C under 3% wet fuel and air electrode atmospheres. At 1.3 V, the PBSCFMNM03 cell delivers higher current densities of 1.88, 1.37, 0.82, and 0.47 A cm^−2^ at 650°C, 600°C, 550°C, and 500°C, respectively, compared to those of the PBSCFMNM01 cell with 1.82, 1.08, 0.55, and 0.22 A cm^−2^ at 1.3 V and the corresponding temperatures. The same defect chemistry that benefits oxygen incorporation in PBSCFMNM03 can also facilitate oxygen evolution and surface exchange under anodic polarization. Thus, the Mo‐enriched electrode provides reversible activity, which is essential for R‐PCECs intended to switch between power generation and electrolysis. The electrolysis mode performance comparison in Figure 5g demonstrates that the PBSCFMNM03 delivers superior electrolysis activity relative to recently reported benchmarks, especially at intermediate‐to‐low temperature. Although the absolute performance of the PBSCFMNM03‐based cell at 600–650°C does not exceed the highest values reported for all R‐PCECs, the cell maintains competitive performance both in fuel cell mode and electrolysis mode as the operating temperature is decreased. This characteristic is particularly relevant to PCECs, whose proton‐conducting electrolytes are intended to enable efficient operation at lower temperatures than conventional oxide‐ion‐conducting solid oxide cells. As temperature decreases, air electrode surface exchange and interfacial charge‐transfer processes become increasingly limiting. The combination of high fuel cell mode power output and high electrolysis current density of PBSCFMNM03 proves its potential as an air electrode for R‐PCECs, which require balanced performance in both directions. The long‐term durability under reversible operation was evaluated by cycling the PBSCFMNM03 cell between fuel cell mode and electrolysis mode conditions at 600°C, as shown in Figure 5h. The cell was alternately operated at 0.8 and 1.2 V for 240 h in wet H_2_ and wet air atmospheres containing 3% H_2_O. The current response remains periodic and relatively stable during the test, with no abrupt failure or progressive collapse visible in the curve. This result indicates that PBSCFMNM03 can withstand repeated changes in electrode polarization and gas‐side reaction direction under the tested conditions. The durability behavior is consistent with the steam‐treated XRD in Figure 3g and the low Sr surface enrichment in Table S7, both of which suggest that PBSCFMNM03 retains structural and surface stability in humid environments, highlighting its strong potential for R‐PCEC applications.

## 3 Conclusion

This study presents PBSCFMNM_x_ perovskite oxides as efficient bifunctional air electrodes for R‐PCEC applications. Crystallographic analyses confirm that PBSCFMNM01 and PBSCFMNM03 crystallize into single‐phase layered double perovskite structures, while atomic‐scale observations verify local crystallinity and homogeneous distribution of Pr, Ba, Sr, Co, Fe, Mn, Ni, Mo and O. Analyses of surface chemistry and hydration also reveal that the higher Mo content significantly increases the concentration of oxygen vacancies, accelerates the oxygen exchange kinetics, and improves hydration behavior. PBSCFMNM03 also exhibits strong chemical stability under H_2_O exposure, proving its chemical robustness. EIS and DRT analyses demonstrate that PBSCFMNM03 exhibits faster ORR kinetics and lower R_p_ than PBSCFMNM01 on both BZCYYb and GDC electrolytes. When implemented in full R‐PCECs, the PBSCFMNM03 air electrode enables improved fuel cell power output (1.10 W cm^−2^) and enhanced electrolysis current density (1.88 A cm^−2^ at 1.3 V) at 650°C, together with stable long‐term reversible operation for over 200 h at 600°C. Mechanistically, the performance enhancement is attributed to Mo‐mediated compositional regulation within the fixed Mn/Ni‐containing PBSCF framework rather than to a meaningful configurational entropy change. Increasing the Mo content modifies local B─O bonding and redistributes the oxidation states of the redox‐active cations, thereby regulating oxygen nonstoichiometry, lattice oxygen lability, hydration response, and charge transport behavior. These coupled changes facilitate oxygen surface exchange, lattice incorporation, and electrode‐electrolyte interfacial charge transfer, resulting in lower polarization resistance and improved reversible cell performance in both fuel cell and electrolysis modes. This work provides useful guidance for the rational design of durable and high‐performance R‐PCEC air electrodes, supporting efficient power generation and hydrogen production through a cost‐effective multi‐element micro‐doping strategy.

## Author Contributions

**Wenkai Yang**: investigation, methodology, software, data curation, formal analysis, validation, Writing – original draft, Writing – review and editing, conceptualization, visualization. **Yue Bao**: validation, writing – review and editing. **Timileyin Aworinde**: investigation, validation, writing – review and editing, software. **Linfeng Yu**: writing – review and editing, validation. **Shaikh Abdullah**: validation, writing – review and editing. **Lakshya Mathur**: validation, writing – review and editing. **Daofan Wang**: validation, writing – review and editing, visualization. **Lourdes F. Vega**: project administration, resources, supervision, funding acquisition, writing – review and editing. **Chuancheng Duan**: project administration, resources, supervision, writing – review and editing, methodology. **Sivaprakash Sengodan**: conceptualization, writing – review and editing, supervision, resources, project administration, funding acquisition, validation, formal analysis, methodology, data curation.

## Funding

This work was supported by the Khalifa University through the faculty start‐up grant under Project ID KU‐INT‐FSU‐2023‐8474000532.

## Conflicts of Interest

The authors declare no conflicts of interest.

## Supporting information




**Supporting File**: advs78109‐sup‐0001‐SuppMat.docx.

## Data Availability

The data that support the findings of this study are available in the supplementary material of this article.
