# Supplementary material for: B‐Site Multi‐Element Micro‐Doping Engineered Air Electrodes With Fast Oxygen Kinetics for Reversible Protonic Ceramic Electrochemical Cells
Source: Adv Sci (Weinh). 2026 Sep 30:e78109. Online ahead of print. doi: 10.1002/advs.78109 (PMC13627772; doi:10.1002/advs.78109)
Supplement: Supplementary file 1 [file ADVS-9999-e78109-s001.docx]

**Supporting Information**

**B-site Multi-element Micro-doping Engineered Air Electrodes with Fast Oxygen Kinetics for Reversible Protonic Ceramic Electrochemical Cells**

*Wenkai Yang^1,2,3^, Yue Bao^3^, Linfeng Yu^3^, Timileyin Aworinde^1,2^, Shaikh Abdullah^1,2^, Lakshya Mathur^1,2^, Daofan Wang^1,2^, Lourdes F. Vega^2,4,5^, Chuancheng Duan^3,*^, Sivaprakash Sengodan^1,2,*^*

*^1^. Department of Mechanical and Nuclear Engineering, Khalifa University, Abu Dhabi, UAE*

*^2^. Research and Innovation Center on CO_2_ and Hydrogen (RICH), Khalifa University, Abu Dhabi, UAE*

*^3^. Department of Chemical Engineering, University of Utah, Salt Lake City, Utah 84112, United States*

*^4^. Department of Chemical and Petroleum Engineering, Khalifa University, Abu Dhabi, UAE*

*^5^. Energy Institute, Khalifa University, UAE*

*^*^ Corresponding Authors’ email addresses:* [*c.duan@utah.edu*](mailto:c.duan@utah.edu)*,* [*sivaprakash.sengodan@ku.ac.ae*](mailto:sivaprakash.sengodan@ku.ac.ae)

**Experimental Section**

Air electrode and electrolyte powder preparation

PrBa_0.5_Sr_0.5_Co_1.5_Fe_0.5_O_5+δ_ (PBSCF), PrBa_0.5_Sr_0.5_Co_1.5_Fe_0.29_Mn_0.1_Ni_0.1_Mo_0.01_O_5+δ_ (PBSCFMNM01), and PrBa_0.5_Sr_0.5_Co_1.5_Fe_0.27_Mn_0.1_Ni_0.1_Mo_0.03_O_5+δ_ (PBSCFMNM03) powders were prepared by the sol-gel method. Stoichiometric amounts of the corresponding metal nitrate precursors including Pr(NO_3_)_3_·6H_2_O, Ba(NO_3_)_2_, Sr(NO_3_)_2_, Co(NO_3_)_3_·6H_2_O, Fe(NO_3_)_3_·9H_2_O, Mn(NO_3_)_2_·4H_2_O, Ni(NO_3_)_2_⋅6H_2_O, (NH_4_)_6_Mo_7_O_24_·4H_2_O for Pr, Ba, Sr, Co, Fe, Mn, Ni, and Mo, respectively, were dissolved in deionized water to obtain a homogeneous precursor solution. Citric acid (CA) and ethylene glycol (EG) were then introduced as complexing and polymerization agents, with a molar ratio of total metal ions:CA:EG = 1:2:3. After complete dissolution, the solution was heated to 300 °C under continuous stirring until a viscous gel was formed and further converted into a black solid precursor. The precursor was subsequently calcined in air at 950 °C for 4 h to obtain the perovskite oxide powders.

BaZr_0.1_Ce_0.7_Y_0.1_Yb_0.1_O_3-δ_ (BZCYYb) and Gd_0.1_Ce_0.9_O_2-δ_ (GDC, Fuel Cell Materials) powders were used for compatibility and electrochemical tests. BZCYYb powder was synthesized by a conventional solid-state reaction. Stoichiometric amounts of BaCO_3_, ZrO_2_, CeO_2_, Y_2_O_3_, and Yb_2_O_3_ were mixed and ball-milled in ethanol for 12 h using zirconia milling media. After drying, the mixed powder was calcined at 1100 °C for 10 h to initiate phase formation, followed by re-milling and a second calcination at 1450 °C for 5 h in air to obtain phase-pure BZCYYb. The resulting powders were ground before X-ray diffraction (XRD) analysis. Chemical compatibility between PBSCFMNM03 and the electrolyte materials was examined using mixed powders of PBSCFMNM03 with BZCYYb or GDC.

Cell fabrication

Dense BZCYYb electrolyte pellets for symmetric cells were fabricated by uniaxially pressing 0.4 g of BZCYYb powder at 5 MPa to form disks with a thickness of approximately 0.5 mm. The brown disks were sintered in air at 1400 °C for 5 h to obtain dense BZCYYb electrolyte substrates. PBSCFMNM_x_ electrode inks were prepared by ball-milling the synthesized PBSCFMNM_x_ powders with an ink vehicle at a mass ratio of 1:0.8 for 24 h. The electrode slurry was screen-printed onto both sides of the electrolyte pellets over a circular active area with a radius of 3 mm and fired at 950 °C for 4 h. Silver paste and silver wires were applied to both air electrode surfaces as current collectors. GDC electrolyte pellets were fabricated by using the same process mentioned above. Symmetric cells with a PBSCFMNM_x_ |electrolyte| PBSCFMNM_x_ configuration were therefore obtained for electrochemical impedance spectroscopy (EIS) measurements.

Anode-supported R-PCEC single cells with a NiO-BZCYYb |BZCYYb| PBSCFMNM_x_ configuration were fabricated through a tape casting-lamination-sintering process. First, a NiO-BZCYYb substrate green tape was prepared by tape casting. A BZCYYb electrolyte brown tape was then tape cast separately. The NiO-BZCYYb substrate tape and BZCYYb electrolyte tape were stacked and laminated using a heated roller to form an integrated multilayer structure. The laminated tapes were punched into cell disks and pre-sintered to remove organic components. The pre-sintered multilayers were subsequently sintered at 1400 °C for 5 h to obtain anode-supported cells with a dense BZCYYb electrolyte layer. Finally, the PBSCFMNM_x_ air electrode slurry was applied to the electrolyte surface and fired at 950 °C for 4 h to complete the R-PCEC single cell fabrication.

Physicochemical characterizations

The phase structure of PBSCF and PBSCFMNM_x_ (x = 0.01 and 0.03) was investigated by XRD (PANalytical Empyrean XRD) utilizing a copper target Kα (α=1.5418) with 40 kV working voltage and 40 mA working current. Rietveld refinement was conducted using BIOVIA Materials Studio software to provide insights into the materials’ crystal structure and lattice parameters. The morphology of air electrode powders and the cross-sectional structure of symmetrical cells and single cells were inspected by scanning electron microscopy (SEM, Quanta 250 FEG ESEM) and high-resolution transmission electron microscopy (TEM, Jem-2100F Jeol). Energy-Dispersive X-ray (EDX) mapping was conducted on Oxford Instruments X-Max 80T. X-ray Photoelectron Spectroscopy (XPS), using a Thermo Fisher Scientific ESCALAB Xi+ system featuring a monochromatic Al Kα X-ray source (1486.6 eV), enabled accurate measurement of binding energies and chemical states of the constituent elements in the air electrode samples. Thermogravimetric analysis (TGA) conducted on NETZSCH STA 449 F3 Jupiter measured the measured the mass changes of the air electrode samples as a function of temperature in dry N_2_, providing insights into the oxidation and reduction behavior through weight changes associated with oxygen loss or uptake. O_2_ temperature-programmed desorption (O_2_-TPD) measurements were carried out using a Micromeritics AutoChem III chemisorption analyzer to evaluate the oxygen desorption behavior of the air electrode powders. For each measurement, approximately 0.15 g of sample was loaded into a quartz reactor. The sample was exposed to an O_2_/He mixture at a flow rate of 20 cm^3^ STP/min for 40 min to achieve oxygen adsorption, followed by purging with He to eliminate physically adsorbed oxygen. The desorption profile was then recorded by heating the sample from ambient temperature to 900 °C at a rate of 10 °C/min under flowing He, while the evolved oxygen species were continuously monitored as a function of temperature. Thermal expansion behavior was measured by dilatometry using a Linseis DIL L75 HS instrument. The PBSCFMNM_x_ powders were pressed into cylindrical specimens. Before each measurement, the initial specimen length, L_0_, was independently determined and used to normalize the recorded dimensional change. The average linear thermal expansion coefficient (TEC) was calculated from the slope of ΔL/L_0_ versus temperature over 25-900 °C.

Electrochemical characterizations

EIS is a commonly used method for probing electrochemical processes such as charge transport, diffusion of ions, and surface reactions, making it ideal for characterizing fuel cell components, including electrodes and electrolytes. EIS measurements were carried out using a BioLogic VSP-3e electrochemical workstation connected to symmetrical cells, covering a frequency range from 100 kHz to 0.1 Hz at an open circuit potential of 30 mV. The measurements began at 650 °C and were performed with a 50 °C decrement until reaching 500 °C. The resulting data were then analyzed and fitted to equivalent circuit models using ZView software.

Before conductivity measurements, the air electrode powders were pelletized into dense cuboid bars and sintered at 1200 °C in air for 10 h. A four-point probe method is used for measurement as it offers more accurate and reliable results than the two-point probe method, which uses one pair of electrodes to apply the current and a separate pair to measure the voltage.

The electrochemical performance of R-PCEC single cells was evaluated in both fuel cell mode and electrolysis mode from 650 to 500 °C with 50 °C decrements. During the tests, humidified H_2_ with 3% H_2_O was supplied to the fuel electrode, while humidified air with 3% H_2_O was supplied to the air electrode. Fuel cell performance was assessed from I-V-P curves, whereas electrolysis performance was evaluated from I-V curves under electrolysis polarization. Reversible cycling stability was further examined at 600 °C under wet H_2_ and wet air conditions to evaluate the operational durability of the R-PCEC single cells.

**Table S1.** Lattice parameters of PBSCFMNM01 and PBSCFMNM03 from Rietveld refinement.

|  | Space group | a (Å) | b (Å) | c (Å) | V(Å^3^) | α (°) | β (°) | γ (°) |
| --- | --- | --- | --- | --- | --- | --- | --- | --- |
| PBSCFMNM01 | P4/mmm | 3.860 | 3.860 | 7.720 | 115.025 | 90 | 90 | 90 |
| PBSCFMNM03 | P4/mmm | 3.858 | 3.858 | 7.716 | 114.864 | 90 | 90 | 90 |

**Table S2.** Fitted binding energies and ratios of O 1*s* of PBSCFMNM01 and PBSCFMNM03 from XPS analysis.

|  | H_2_O | at.% | O_ads_ | at.% | O_lat_ | at.% | O_ads_/O_lat_ |
| --- | --- | --- | --- | --- | --- | --- | --- |
| PBSCFMNM01 | 532.70 | 11.46 | 531.23 | 36.98 | 528.99 | 51.56 | 0.72 |
| PBSCFMNM03 | 532.60 | 10.90 | 530.88 | 38.15 | 528.81 | 50.95 | 0.75 |

**Table S3.** Fitted binding energies and ratios of Co 2*p* of PBSCFMNM01 and PBSCFMNM03 from XPS analysis.

|  | Co^3+^ | | at.% | Co^4+^ | | at.% |
| --- | --- | --- | --- | --- | --- | --- |
|  | Co 2*p_1/2_* | Co 2*p_3/2_* |  | Co 2*p_1/2_* | Co 2*p_3/2_* |  |
| PBSCFMNM01 | 793.76 | 778.50 | 45.82 | 796.11 | 780.85 | 54.18 |
| PBSCFMNM03 | 793.74 | 778.52 | 50.63 | 796.15 | 780.89 | 49.37 |

**Table S4.** Fitted binding energies and ratios of Fe 2*p* of PBSCFMNM01 and PBSCFMNM03 from XPS analysis.

|  | Fe^3+^ | | at.% | Fe^4+^ | | at.% |
| --- | --- | --- | --- | --- | --- | --- |
|  | Fe 2*p_1/2_* | Fe 2*p_3/2_* |  | Fe 2*p_1/2_* | Fe 2*p_3/2_* |  |
| PBSCFMNM01 | 722.50 | 709.70 | 52.34 | 724.72 | 711.92 | 47.66 |
| PBSCFMNM03 | 722.48 | 709.68 | 51.68 | 725.29 | 711.90 | 48.32 |

**Table S5.** Fitted binding energies and ratios of Pr 3*d* of PBSCFMNM01 and PBSCFMNM03 from XPS analysis.

|  | Pr^3+^ | | at.% | Pr^4+^ | | at.% |
| --- | --- | --- | --- | --- | --- | --- |
|  | Pr 3*d_3/2_* | Pr 3*d_5/2_* |  | Pr 3*d_3/2_* | Pr 3*d_5/2_* |  |
| PBSCFMNM01 | 948.50 | 928.08 | 32.74 | 953.26 | 932.65 | 67.26 |
| PBSCFMNM03 | 948.38 | 927.98 | 31.30 | 953.10 | 932.61 | 68.70 |

**Table S6.** Fitted binding energies and ratios of Mn 2*p* of PBSCFMNM01 and PBSCFMNM03 from XPS analysis.

|  | Mn^3+^ | | at.% | Mn^4+^ | | at.% |
| --- | --- | --- | --- | --- | --- | --- |
|  | Mn 2*p_1/2_* | Mn 2*p_3/2_* |  | Mn 2*p_1/2_* | Mn 2*p_3/2_* |  |
| PBSCFMNM01 | 652.80 | 641.44 | 81.02 | 656.06 | 644.26 | 18.98 |
| PBSCFMNM03 | 652.55 | 641.54 | 79.56 | 656.50 | 644.79 | 20.44 |

**Table S7.** Fitted binding energies and ratios of Sr 3*d* of PBSCFMNM01 and PBSCFMNM03 from XPS analysis.

|  | Lattice Sr | | at.% | Surface Sr | | at.% | Surface Sr/Lattice Sr |
| --- | --- | --- | --- | --- | --- | --- | --- |
| PBSCFMNM01 | 133.55 | 131.85 | 59.21 | 135.20 | 133.50 | 40.79 | 0.689 |
| PBSCFMNM03 | 133.40 | 131.85 | 59.60 | 135.10 | 133.35 | 40.40 | 0.678 |

**Figure S1.** XRD patterns of PBSCFMNM03-BZCYYb and PBSCFMNM03-GDC mixtures after calcination at 1100 ℃ for 20 h.

**Figure S2.** XPS spectra of PBSCFMNM01 and PBSCFMNM03.

**Figure S3.** Co 2*p* XPS spectra and peak deconvolution of PBSCFMNM01 and PBSCFMNM03.

**Figure S4.** Fe 2*p* XPS spectra and peak deconvolution of PBSCFMNM01 and PBSCFMNM03.

**Figure S5.** Pr 3*d* XPS spectra and peak deconvolution of PBSCFMNM01 and PBSCFMNM03.

**Figure S6.** Mn 2*p* XPS spectra and peak deconvolution of PBSCFMNM01 and PBSCFMNM03.

**Figure S7.** Sr 3*d* XPS spectra and peak deconvolution of PBSCFMNM01 and PBSCFMNM03.

**Figure S8.** TEC comparison of PBSCFMNM_x_ with other PrBaCo_2_O_5+δ_-based air electrodes [1-7].

**Figure S9.** Temperature-dependent Nyquist plots of BZCYYb electrolyte-supported symmetric cells with PBSCFMNM01 and PBSCFMNM03 air electrodes from 500 to 650 °C.

**Figure S10.** Nyquist plots and corresponding distribution of relaxation times (DRT) analysis of BZCYYb electrolyte-supported symmetric cells with PBSCFMNM01 and PBSCFMNM03 air electrodes at 650 °C, 600 °C, and 500 °C.

**Figure S11.** Temperature-dependent Nyquist plots of GDC electrolyte-supported symmetric cells with PBSCFMNM01 and PBSCFMNM03 air electrodes from 500 to 650 °C.

**Figure S12.** Nyquist plots and corresponding DRT analysis of GDC electrolyte-supported symmetric cells with PBSCFMNM01 and PBSCFMNM03 air electrodes at 650 °C, 600 °C, and 500 °C.

**Figure S13.** I-V-P curve of R-PCEC with PBSCFMNM01 air electrode at various temperatures in fuel cell mode.

(a)

(b)

**Figure S14.** (a) Temperature-dependent Nyquist plots of R-PCECs using PBSCFMNM01 and PBSCFMNM03 air electrodes from 500 to 650 °C. (b) Enlarged high-frequency regions of the above Nyquist plots.

**Figure S15.** Nyquist plots and corresponding DRT analysis of R-PCEC single cells with PBSCFMNM01 and PBSCFMNM03 air electrodes from 500 to 650 °C.

**Figure S16.** I-V curve of R-PCEC with PBSCFMNM01 air electrode at various temperatures in electrolysis mode.

**References**

[1] H. Bai, J. Chu, T. Chen, Z. Leng, B. Zhang, Y. Zhang, Q. Zhou, J. Zhou, S. Wang, PrBa0.5Sr0.5Co1.5Fe0.5O5+δ as air electrode for proton-conducting solid oxide cells, Journal of Power Sources, 574 (2023) 233162.

[2] Y. Gao, M. Fu, S. Zhao, Z. Hou, K. Liu, X. Deng, Z. Tao, Self-Assembled Composite Cathodes with TEC Gradient for Proton-Conducting Solid Oxide Fuel Cells, Advanced Functional Materials, 35 (2025) 2416625.

[3] Q. Guo, S. Li, S. An, R. Sun, M. Ren, L. Gao, Z. Yan, H. Cao, Modification of B-site substituted by high-valence Nb in PrBa0.5Sr0.5Co1.5Fe0.5O5+δ as a highly active air electrode for solid oxide fuel cell, RSC Advances, 15 (2025) 9110–9118.

[4] F. He, Y. Zhou, T. Hu, Y. Xu, M. Hou, F. Zhu, D. Liu, H. Zhang, K. Xu, M. Liu, Y. Chen, An Efficient High-Entropy Perovskite-Type Air Electrode for Reversible Oxygen Reduction and Water Splitting in Protonic Ceramic Cells, Advanced Materials, 35 (2023) 2209469.

[5] S. Oh, I. Jeong, D. Kim, H. Kim, K.-M. Roh, K.T. Lee, Unveiling Entropy-Driven Performance Enhancement in Double Perovskite Oxygen Electrodes for Protonic Ceramic Electrochemical Cells, Advanced Energy Materials, 15 (2025) e03176.

[6] Y. Xu, K. Xu, F. Zhu, F. He, H. Zhang, C. Fang, Y. Liu, Y. Zhou, Y. Choi, Y. Chen, A Low-Lewis-Acid-Strength Cation Cs+-Doped Double Perovskite for Fast and Durable Oxygen Reduction/Evolutions on Protonic Ceramic Cells, ACS Energy Letters, 8 (2023) 4145–4155.

[7] L. Zhao, Q. Nian, B. He, B. Lin, H. Ding, S. Wang, R. Peng, G. Meng, X. Liu, Novel layered perovskite oxide PrBaCuCoO5+δ as a potential cathode for intermediate-temperature solid oxide fuel cells, Journal of Power Sources, 195 (2010) 453–456.
